# Cholera Epidemics, War and Disasters around Goma and Lake Kivu: An Eight-Year Survey

**DOI:** 10.1371/journal.pntd.0000436

**Published:** 2009-05-19

**Authors:** Didier Bompangue, Patrick Giraudoux, Martine Piarroux, Guy Mutombo, Rick Shamavu, Bertrand Sudre, Annie Mutombo, Vital Mondonge, Renaud Piarroux

**Affiliations:** 1 Direction de la Lutte contre les Maladies, Ministère de la Santé Publique, Kinshasa-Gombe, République Démocratique du Congo; 2 Laboratoire Chrono-Environnement, CNRS, UMR 6249, Université de Franche-Comté, UFR Sciences et Techniques, Besançon, France; 3 Service de Microbiologie, Université de Kinshasa, Kinshasa, République Démocratique du Congo; 4 Laboratoire Théoriser et Modéliser pour Aménager (ThéMA), UMR 6049 du CNRS, Université de Franche-Comté, Besançon, France; 5 Division Provinciale de la Santé-Nord Kivu, Ministère de la Santé Publique, Goma, République Démocratique du Congo; 6 Division Provinciale de la Santé-Sud Kivu, Ministère de la Santé Publique, Uvira, République Démocratique du Congo; Massachusetts General Hospital, United States of America

## Abstract

**Background:**

During the last eight years, North and South Kivu, located in a lake area in Eastern Democratic Republic of Congo, have been the site of a major volcano eruption and of numerous complex emergencies with population displacements. These conditions have been suspected to favour emergence and spread of cholera epidemics.

**Methodology/Principal Findings:**

In order to assess the influence of these conditions on outbreaks, reports of cholera cases were collected weekly from each health district of North Kivu (4,667,699 inhabitants) and South Kivu (4,670,121 inhabitants) from 2000 through 2007. A geographic information system was established, and in each health district, the relationships between environmental variables and the number of cholera cases were assessed using regression techniques and time series analysis. We further checked for a link between complex emergencies and cholera outbreaks. Finally, we analysed data collected during an epidemiological survey that was implemented in Goma after Nyiragongo eruption. A total of 73,605 cases and 1,612 deaths of cholera were reported. Time series decomposition showed a greater number of cases during the rainy season in South Kivu but not in North Kivu. Spatial distribution of cholera cases exhibited a higher number of cases in health districts bordering lakes (Odds Ratio 7.0, Confidence Interval range 3.8–12.9). Four epidemic reactivations were observed in the 12-week periods following war events, but simulations indicate that the number of reactivations was not larger than that expected during any random selection of period with no war. Nyiragongo volcanic eruption was followed by a marked decrease of cholera incidence.

**Conclusion/Significance:**

Our study points out the crucial role of some towns located in lakeside areas in the persistence of cholera in Kivu. Even if complex emergencies were not systematically followed by cholera epidemics, some of them enabled cholera spreading.

## Author Summary

With the number of cholera cases up to 73,000 during the last eight years and successive wars that have persisted for fifteen years, the North and South Kivu provinces of the Democratic Republic of Congo are currently heavily hit by both cholera outbreaks and war-related population displacements. Prior to this study, no research had been done to identify the sources of epidemics and the pathways used by cholera to spread throughout the Kivu provinces. Here we show that a few cities located on the lakeshore of Lake Kivu and Lake Tanganyika act as the main sources of cholera epidemics and that the number of cholera cases tends to increase during the rainy season. We also found that only a minority of population displacements were followed by cholera outbreaks. Finally, we think that the low number of cholera cases recorded after the Nyiragongo eruption is one more argument to implement programs aiming at restoring, and if possible improving, drinking water access following natural disasters

## Introduction

Numerous factors have been postulated to increase the risk of cholera outbreaks in a given area where cholera is already circulating among the population. The main environmental risk factors identified include heavy rainfall, blooms of plankton, and an increase in sea surface temperatures [1]. However, most studies have been performed in coastal areas and very little is known about environmental factors involved in the recurrence of cholera epidemics in inland areas. In this context, it has been recently shown that the lake areas have been the source of iterative cholera outbreaks in the inland areas of Katanga, a province located south-east of the Democratic Republic of Congo (DRC) [2]. Deadly cholera outbreaks have also been reported during complex emergencies (CEs) that are defined as “a humanitarian crisis in a country, region or society where there is a total or considerable breakdown of authority resulting from internal or external conflict, and which requires an international response that goes beyond the mandate or capacity of any single and/or ongoing United Nations (UN) country programme” [3]. Lastly, probably by analogy with CEs, the risk of cholera epidemics is also an often-repeated assertion in the aftermath of large-scale natural disasters [4].

The provinces of North and South Kivu, bordering Lake Kivu in the east of the DRC, present an exceptional accumulation of these risk factors. They have been the site of numerous dramatic events, including invasion and occupation by foreign forces, civil war, population displacements, a major volcano eruption and earthquakes. According to a recently published mortality survey, it is estimated that the conflicts and humanitarian crises, which ravaged the eastern part of the DRC, have taken the lives of 5.4 million people since 1998, and continue to leave as many as 45,000 dead every month [5]. Despite the tragic events encountered in the Kivu provinces, an epidemiological surveillance system was set up at the end of the 1990's, and it is still recording data on cholera and other communicable diseases.

Here, we present a study designed to describe epidemiological patterns of cholera outbreaks in the Kivu provinces and to elucidate the influence of specific environmental and geographical factors, CEs and disasters on outbreaks of cholera. This study and the previous work that described the patterns of cholera outbreaks in Katanga and Eastern Kasaï [2] constitute the first step of a plan aiming to fight cholera in the DRC.

## Methods

From January 2000 through December 2007, reports of cholera cases and deaths were collected weekly from each health district (HD) of North and South Kivu provinces. Case-patients of cholera were defined as recommended by the World Health Organization (WHO): “any person 5 years of age or older in whom severe dehydration develops or who dies from acute watery diarrhoea”, with an age limit lowered to 2 years for cases associated with confirmed cholera outbreaks [6]. Also as recommended by the WHO, each new important outbreak was confirmed by culture and identification of *Vibrio cholera* O1 from stool samples [6].

North Kivu (53,855 km^2^, 4,667,699 inhabitants, 19 HDs) and South Kivu (65,000 km^2^, 4,670,121 inhabitants, 14 HDs) are located in the Great Rift Valley, and border Lake Edward, Lake Kivu, and the north edge of Lake Tanganyika. In Kivu, the climate is characterized by a rainy season from October to the end of May and a dry season the rest of the year. However, the rainy season is partially interrupted by a short dryer period in January and February. The relief of the Kivu provinces is dominated by several volcanic chains. Nyiragongo, the most active volcano, is located approximately 20 kilometres north of the city of Goma (400,000 inhabitants) near Lake Kivu. Nyiragongo last major eruption occurred on January 17, 2002, when lava flow destroyed one third of the city of Goma [7], claiming 147 victims (Didier Bompangue, personal data). In response to this disaster, the international community brought quick and massive help to the population by providing safe drinking water. Moreover, during the 12-week period following the disaster, access to health care facilities was improved, due to the humanitarian response. In particular, a program of drug supply was implemented to support the primary health centres in Goma and health facilities were made free for a six-week period, followed by another six-week period with reduced prices (0.2 $ instead of 1 $ for ambulatory care services, including drugs). Reports on CEs which occurred in the Kivu provinces from 2000 to 2007 were collected from the Reliefweb Website, which compiles information from a variety of sources, including UN Agencies and non-governmental organizations (NGOs) [7]. Among them, we further selected the CEs which were subjected to a medical assessment by humanitarian organizations and which involved more than 1000 internally displaced persons (IDPs).

A geographic information system was established, based on the data collected from the 33 HDs of the two provinces. Following a previously described procedure [2], we statistically examined the relationship between the number of cholera cases in each HD and geographic and environmental variables (area, population, and presence/absence of cities with >100,000 inhabitants, of at least one commercial port, of major tracks or roads, and of lakeside location for each HD). Population and area were log transformed and log(population) included as an offset term in the model. Due to the overdispersion of cholera incidence, several kinds of generalized linear models were compared using quasi-Poisson, and type I and type II negative binomial distributions and they were checked for spatial structure. Stepwise selection of variables was performed in each case and the best models of each family were compared using the Akaïke index criterion, according to Venables and Ripley [8] and Rigby et al. [9] The relationship between the number of cholera cases in health districts and geographical variables was finally modelled using the type II negative binomial family (log link function for both the mean and the distribution parameter). The residuals were checked for spatial structure by plotting an empirical variogram where the distances were computed depending on the geographical coordinates of the centroid of each health district. A variogram envelope was then computed by performing 1000 permutations of the residual values on the spatial locations and the envelope limits were then compared to the variogram. In the present study, we failed to detect residual spatial autocorrelation.

The rate of the cholera cases was mapped for the 33 HDs using ESRI shapefiles. Cross-correlations between time-series of HDs were computed [10]. Time series, which were synchronous (i.e. with no time lag) in a geographical area, were merged. This led to define 5 zones (zone 1: Mutwanga; zone 2: Goma, Kirotshe; zone 3: Bukavu, Katana; zone 4: Uvira, Nundu, Fizi; zone 5: Pinga, Walikale). The time-series obtained were decomposed into a trend, a seasonal component and a remainder using a seasonal-trend decomposition procedure based on loess regression (STL) following Cleveland et al. (1990) [11]. In time series analysis, non-parametric STL methods have the advantage of robustness and simplicity but do not allow predictions and detailed quantification of time-series parameters, not necessary for our purpose in the present study. The remainder was examined and, each week, zones with an above-average number of cases were marked as an epidemic reactivation. If epidemic reactivation was typically fostered by war events in a non-epidemic period, one would expect more epidemic reactivations within the 12 weeks following a war event than within the 12 weeks following any randomly selected no-war week. This hypothesis was tested on a basis of 1000 simulations, checking for the occurrence of at least one (or the absence of any) epidemic reactivations during the 12 weeks following each week randomly selected during a non-epidemic period. The number of randomly selected weeks considered was proportional to the number of war events actually observed in each zone. The impact of the Nyiragongo 2002 eruption was also analysed in relation to the dynamics of cholera epidemics. In addition, during the 12-week period following the eruption of Nyiragongo, an epidemiological survey was implemented in primary care patients of Goma. During this period, all cases of acute diarrhoea, upper and lower respiratory tract infection and fever were recorded from five local health centres located within the western area of Goma. These data were compared to the records of the centres corresponding to the three weeks before the disasters.

Computing and graphical displays were done using ArcGIS 8.3 and R 7.2 [12], and the following additional packages: MASS version 44 [8], maptools [13], sp [14], GAMLSS [15], and geoR [16].

## Results

From January 2000 through December 2007 (416 weeks), a total of 73,605 cases and 1,612 deaths (lethality: 2.2%) from cholera were reported in North and South Kivu. *Vibrio cholerae* O1 El Tor Ogawa was isolated in 8 samples collected from North Kivu (among 38 samples) and 3 from South Kivu (among 29 samples). *Vibrio cholerae* O1 El Tor Inaba was found only in South Kivu (6 positive samples among 29). In Bukavu (South Kivu), Ogawa and Inaba serotypes were isolated during the same outbreak of cholera in 2005. All isolates were found to be sensitive to ciprofloxacin, erythromycin and nalidixic acid, and resistant to tetracycline, ampicillin and cotrimoxazole.

**Figure 1:**
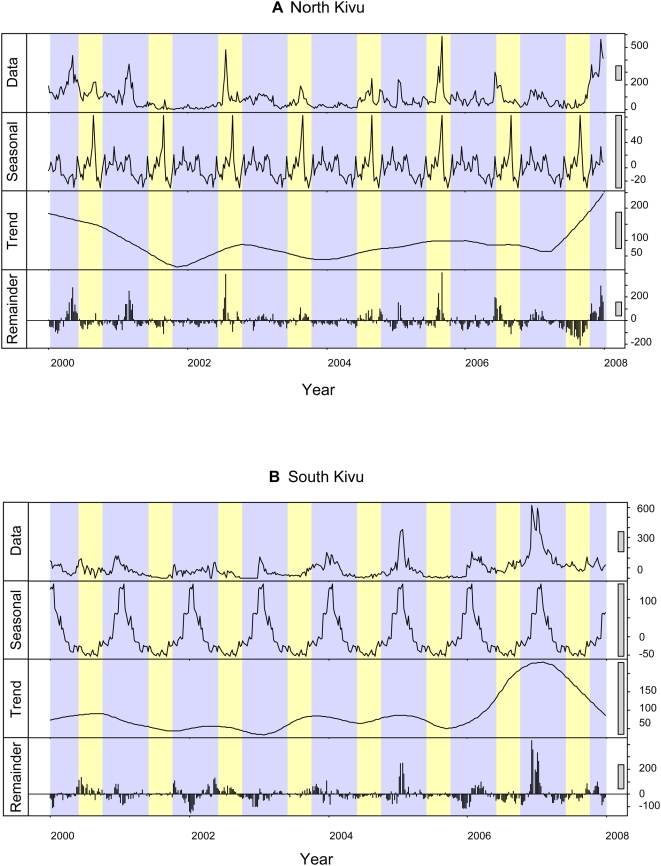
Time-series of the number of weekly cholera cases from 2000 to 2007. A: North Kivu and B: South Kivu; yellow: dry season, blue: rainy season. Time-series (data, weekly number of cases) are broken down into seasonal, trend and remainder components. Seasonal, trend and remainders sum to the weekly number of cases (data).

**Figure 2:**
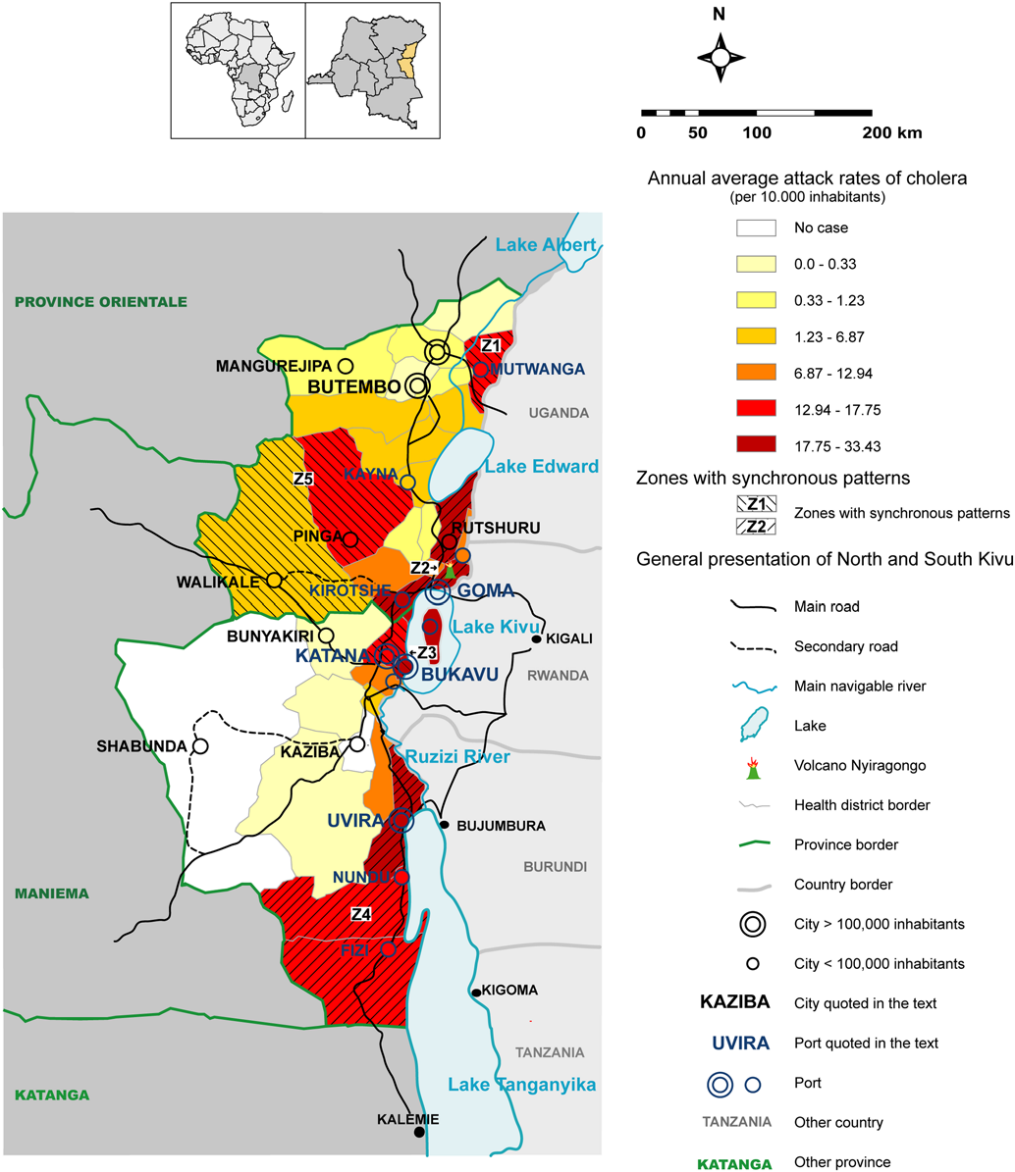
Distribution of cholera in North and South Kivu from 2000 through 2007. Average annual attack rate of cholera per 10,000 inhabitants, in each health district.

During the eight-year study, both provinces experienced at least one outbreak of cholera per year, with peaks ranging from 130 to more than 700 cases a week (Figure 1). In South Kivu, cholera cases were reported in every week except for two short periods in 2001 and 2002 (Figure 1). Time-series decomposition showed a marked seasonal influence, with a greater number of cases during the rainy season. In North Kivu, no period without cholera could be identified. Seasonal patterns were notably different in North Kivu from those in South Kivu, with epidemics occurring during both dry and rainy seasons (Figure 1). Occasionally, periods of partial remission occurred, during which the weekly incidence of the disease was below 1/50,000 inhabitants (in 2001, weeks 29, 30, 31, 32, 33, 35 and 40, in 2004, weeks 23 and 28). Each time, cholera epidemics again appeared, stemming from residual cases located in Goma (North Kivu) and Uvira (South Kivu). The spatial distribution of cholera cases was heterogeneous, with a higher number of cases in HDs bordering lakes whereas two remote HDs, Kaziba and Shabunda reported no cases of cholera (Figure 2). Table 1 shows that the number of cholera cases was significantly higher in the presence of a lake (odds ratio [OR] 7, 95% confidence interval range [CI] 3.8–12.9).

**Table 1:** Model parameters and odds ratios of the negative binomial model selected.

	Relative risk	Lower limit 95% CI^1^	Upper limit 95% CI^1^
Intercept^2^	0.001	0.001	0.002
City over 100,000 inhabitants	1.464	0.746	2.873
Port	1.608	0.687	3.765
Lake	7.015	3.813	12.904

Suspected cholera cases in North Kivu and South Kivu, Democratic Republic of Congo, 2000–2007.

1 CI, confidence interval.

2 Intercept, average number of cases per health zones.

**Figure 3:**
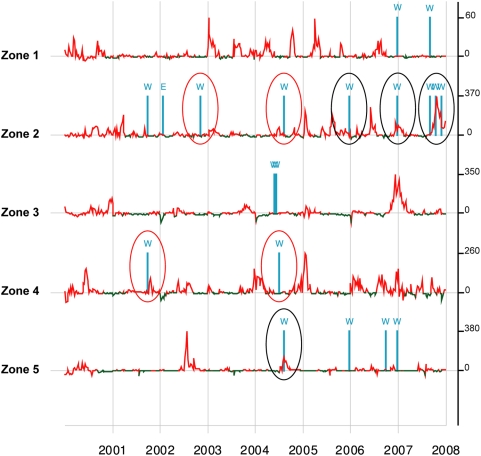
Cholera outbreaks and main events in the 5 synchronous sets (Z) of health districts. War events (W) and the main natural disaster (Nyiragongo eruption- E) observed in North and south Kivu from 2000 to 2007. Zone 1: Mutwanga; Zone 2: Goma, Rutshuru, Kirotshe; Zone 3: Bukavu, Katana. Zone 4: Uvira, Nundu, Fizi; Zone 5: Pinga, Walikale. The vertical axis represents the remainders of the time-series (this removes the undesirable effects of seasonal and inter-annual trends; in red: the periods above average; in green: those below average). War events, which occurred during an already occurring outbreak, are circled in black. War events followed by cholera outbreak starting within 12 weeks are circled in red.

According to UN and NGOs reports, a total of 18 large-scale population displacements related to CEs has been recorded during the period. In six cases, these population displacements occurred during already ongoing cholera epidemics. Among the 12 remaining war events with displacements of population, four were followed by a cholera outbreak within a period of 12 weeks. Two of these cholera epidemics occurred in IDP camps, starting six and eight weeks after the arrival of the first IDPs in the settlements. However, simulations indicate that the number of reactivations was not larger than expected after any random selection of a week with no war event in a non-epidemic period (Figure 3).

In 2002, the Nyiragongo volcanic eruption was not followed by any exacerbation of cholera incidence. A survey performed in five health centres located in the western area of Goma showed that, during this period, diarrhoeas accounted for only 6% of patients who were seen in the health centres. In the entire city of Goma, only 140 cholera cases (8 cases per week), without any deaths, were reported from January to April 2002. This low number of cases contrasts with an average of 29 cases per week usually encountered in Goma at this period of the year.

## Discussion

Since the beginning of the 1990's, Kivu provinces have been identified as one of the most active foci of cholera in the world. In 1994, the refugee camps located around Goma and Bukavu experienced the deadliest cholera epidemics recorded during the last hundred years. This explosive outbreak of cholera, which affected Rwandan refugees, resulted in about 70,000 cases and 12,000 deaths [17]. Since that period, cholera epidemics have been common in the Kivu provinces, and, in a review of reported cholera outbreaks worldwide, from 1995 to 2005, Griffith et al. notes that the eastern DRC provinces are among the most affected zones in Africa [18]. Notwithstanding the inherent limitations associated with epidemiological data collected in developing countries, especially in a context of civil war, our study is the first that provides spatial data collected weekly during an eight-year period for a population of ten million living in an area severely affected by cholera. Even though the number of cases reported in this study is impressive (more than 73,000 cases), it is likely to be underestimated due to the situation of insecurity which prevailed in this region during the studied period, and which led to poor access to health facilities. By contrast, one cannot exclude that some patients suffering from other diarrhoeal diseases may have been included in the study: indeed, only a few clinical cases have been confirmed by culture, even if that may be due to inappropriate sampling procedures (samples collected from buckets containing chlorine) as well as an excessive delay to handle the samples to the laboratories. Moreover, given the lack of local laboratory facilities, other causes of acute watery diarrhoea could unfortunately not be investigated. For instance, in Bangladesh a number of epidemics of watery diarrhoea are actually caused by ETEC (Enterotoxigenic *Escherichia coli*), which would have a similar clinical presentation to that of the cholera cases here [19]. Therefore, our results obtained using a clinical definition of cases, represent only an estimate of the real cholera burden in the Kivu provinces. Nevertheless, we believe our results to highlight some significant aspects of cholera epidemiology that need further discussion.

First, our findings, obtained from a study performed in a lakeside region, are consistent with what was recently found in the province of Katanga, located on the south side of Kivu in the eastern part of the DRC [2]. This previous study showed that 60 percent of cases that were observed in Katanga and Eastern Kasaï between 2002 and 2005 actually occurred in a few lake areas. In these two provinces, the number of cholera cases was significantly higher in the presence of a lake, (OR 7.5, 95% CI: 3.9–14.2). The present study confirms that the same trends can be observed in the Kivu Provinces. Similar to the role played by the towns of Kalemie and Bukama in Katanga, the cities of Goma, Bukavu and Uvira seem to act as the main sources of cholera epidemics in the Kivu provinces. From an operational point of view, this finding implies that more attention should be paid to cholera in these towns, especially in periods when outbreaks are starting with a small, but rising number of cases.

Here, the influence of seasons, and the effect that some lakeside areas have on the persistence of the disease, could make the epidemiological pattern of cholera in Kivu comparable to patterns studied more comprehensively in Asian coastal areas [1],[20],[21]. Two studies have stressed the link between having cholera and living on the shores of a lake or a river, which includes drinking the water and bathing in it. One of these studies was carried out in Rumonge, a city in Burundi bordering Lake Tanganyika, the same lake where the port of Uvira is located [22]. Another one, carried out in nearby Lake Victoria in Kenya, suggested the possible existence of at least a transient environmental reservoir for cholera in the lake and evoked the possible role of water hyacinths in maintaining environmental sources of toxigenic cholera strains during inter-epidemic periods.[23]. However, lake water differ from estuarine brackish water that is known to be the natural reservoir of *V. cholerae* [24]–[26]. Even though lake water can sometimes be rich in plankton [27], the role of lakes as reservoirs for *V. cholerae* is not formally established because no study has yet demonstrated a long term persistence of toxigenic *V. cholerae* in the East African Rift Valley lakes. Our results show that there is a need for further studies to explore the role of lake environments in the persistence of cholera in inland Africa. Indeed, some endemic *V. cholerae* strains have long been isolated from fresh water [28] and Kirschner et al. recently demonstrated the permanent existence of non-toxigenic *V. cholerae* strains, which can rapidly grow in a free-living state in one natural lake water in Austria [29].

The eruption of Nyiragongo, the largest natural disaster reported during this period, was not followed by a cholera outbreak, or by any other disease outbreak. On the contrary, during the months that followed the disaster, the rates of cholera infection were among the lowest that have been recorded in the city of Goma during the 8 years of the study. Several hypotheses can be advanced to explain this low number of cholera cases. This can be due to the impact of the emergency program, but other possible explanations cannot be ruled out, including the fact that the volcanic eruption could have decreased the likelihood of a cholera outbreak secondary to the alterations in water sources and usage patterns. Our finding is in agreement with a recent study showing that earthquakes, tsunamis and volcano eruption were not usually followed by epidemics [30]. In particular, for 20 years, not a single cholera outbreak has been recorded in the aftermath of geophysical disaster, even after the cataclysmic tsunami in Asia in 2004. Here, we show that even in a place where cholera outbreaks are common and during a period known to be favourable for epidemics (the rainy season), a disaster that destroyed approximately 12,000 homes and partially destroyed the water supply pipelines of a town of 400,000 inhabitants, the occurrence of cholera epidemics was not unavoidable.

The search for the impact of the CEs indicates that they do not systematically represent a triggering factor for cholera outbreaks. However, in our study, we also saw that in four cases, the occurrence of a CE led to the exacerbation of cholera, including, in two cases a cholera outbreak, which started in a displaced settlement a few weeks after the arrival of IDPs. Actually, several conditions are necessary for a CE to father a cholera outbreak. Among these conditions, there is the fact that some of the IDPs fleeing from the conflict area should have a previously acquired cholera infection (symptomatic or in incubation), and/or the fact that the IDPs should move into areas where cases of cholera are already present. It would also be necessary for there to be insufficient or no assistance to the IDPs (i.e. provisions for safe drinking water and a system of free health care). These circumstances were met in 1994 when one million refugees coming from Rwanda settled in makeshift camps around Goma, overwhelming the capacities of humanitarian staff already present in the town. More recently, due to the insecurity that prevailed around Goma in the summer and fall of 2008, a cascade of cholera outbreaks that began in Rutshuru in the North of Goma have been recorded in North Kivu: Rutshuru (beginning on week 37), Goma and Karisimbi (beginning on week 40), Walikale and Birambizo (beginning on week 44). In each of these HDs, the outbreak was introduced by people escaping from battle-hit areas located North of Goma (D. Bompangue, personal data). Simultaneously, due to excessive danger for fieldworkers, numerous NGOs fled from Goma and neighbouring areas as cholera outbreaks started, leading to a disorganization of the programs aimed to limit the spread of the epidemic.

In conclusion, the epidemiology of cholera in both Kivu provinces confirms our previous findings in Katanga and eastern Kasaï, and highlights the role of some towns located in lakeside areas as sources of cholera outbreaks. The results of this study show that, even if each CE with numerous IDPs is not systematically followed by a cholera outbreak, CEs may facilitate spreading of already existing outbreaks due to the fleeing of infected IDPs to new areas where NGOs cannot reach them due to an excessive danger for fieldworkers. By contrast, even in a context of CE and natural disaster, the occurrence of epidemics is not unavoidable. For example, the number of cholera cases was lower than expected after the partial destruction of the town by the Nyiragongo eruption followed by the implementation of an emergency program. We think that this low number of cholera cases is one more argument to implement programs aiming to restore, and if possible to improve, drinking water access following natural disasters.

## Supporting Information

Alternative Language Article S1 Translation of the Article into French.
(0.96 MB PDF)
