# Supplementary material for: Cholera Epidemics, War and Disasters around Goma and Lake Kivu: An Eight-Year Survey
Source: PLoS Negl Trop Dis. 2009 May 19;3(5):e436. doi: 10.1371/journal.pntd.0000436 (PMC2677153; doi:10.1371/journal.pntd.0000436)
Supplement: Alternative Language Article S1 — Translation of the Article into French. (0.96 MB PDF) [file pntd.0000436.s001.pdf]

Epidémies de choléra, guerre et catastrophes dans la région de Goma et du lac Kivu : une étude sur huit années.

Auteurs et affiliations

Didier Bompangue,[1,2,3] Patrick Giraudoux,[2] Martine Piarroux,[4] Guy Mutombo,[5] Rick Shamavu,[6] Bertrand Sudre,[2] Annie Mutombo,[1] Vital Mondonge,[1] Renaud Piarroux.*[2]

[1] Direction de la Lutte contre les Maladies, Ministère de la Santé Publique, Kinshasa-Gombe, République Démocratique du Congo.

[2] Laboratoire Chrono-Environnement, CNRS, UMR 6249, Université de Franche-Comté, UFR Sciences et Techniques, 25030 Besançon, France.

[3] Service de microbiologie, Université de Kinshasa, RDC.

[4] Laboratoire Théoriser et Modéliser pour Aménager (ThéMA), UMR 6049 du CNRS, Université de Franche-Comté, 25030, Besançon, France.

[5] Division Provinciale de la Santé-Nord Kivu, Ministère de la Santé Publique, République Démocratique du Congo.

[6] Division Provinciale de la Santé-Sud Kivu, Ministère de la Santé Publique, République Démocratique du Congo.

***Auteur correspondant:** Renaud Piarroux renaud.piarroux@ap-hm.fr Tel :+ 33491387558

Résumé

Contexte Au cours de ces huit dernières années, le Sud et le Nord Kivu, situés dans une région de lacs dans l'est de la République Démocratique du Congo, ont été touchés par une éruption volcanique majeure et par de nombreuses situations de crises humanitaires avec des déplacements de populations. Ce type de situation est considéré comme favorable à l'émergence et à la diffusion des épidémies de choléra.

Méthodologie/principaux résultats Afin d'évaluer l'influence de ces conditions sur les épidémies, les cas de choléra ont été recensés semaine par semaine pour chaque zone de santé du Nord (4 667 699 habitants) et du Sud Kivu (4 670 121 habitants) de 2000 à 2007 inclus. Un système d'information géographique a été créé et, dans chaque zone de santé, les relations entre variables environnementales et nombre de cas de choléra ont été évaluées à l'aide d'analyses de régression numérique et de séries temporelles. Nous avons ensuite recherché un lien entre les crises humanitaires et les épidémies de choléra. Enfin, nous avons analysé les données collectées lors de la surveillance épidémiologique effectuée à Goma après l'éruption du volcan Nyiragongo.

Au total, 73 605 cas et 1612 décès par choléra ont été notifiés. La décomposition en séries temporelles fait apparaître un excédent de cas pendant la saison des pluies au Sud Kivu mais pas au Nord Kivu. La distribution spatiale des cas de choléra met en évidence un nombre de cas plus élevé dans les zones de santé situés au bord d'un lac (Odds Ratio 7,0, avec un intervalle de confiance à 95% de 3,8 à 12,9). Quatre réactivations épidémiques ont été observées pendant une période de 12 semaines à la suite d'un épisode de combat, mais les simulations ont montré que ce nombre n'est pas supérieur à celui obtenu après n'importe quelle sélection randomisée de périodes sans combat. L'éruption du volcan Nyiragongo fut quant à elle suivie d'une diminution marquée de l'incidence du choléra.

Conclusion Notre étude souligne le rôle prépondérant de certaines villes situées en bord de lac dans la persistance du choléra au Kivu. Même si les crises hmanitaires ne sont pas systématiquement suivies d'épidémies de choléra, certaines ont pu faciliter leur diffusion.

Résumé de l'auteur

Avec un nombre de cas de choléra supérieur à 73 000 au cours de ces huit dernières années, et les guerres qui se sont succédées depuis quinze ans, les provinces du Nord et du Sud Kivu en République Démocratique du Congo sont encore actuellement lourdement frappées à la fois par les épidémies de choléra et par les crises humanitaires entraînant des déplacements de population. Avant ce travail, aucune étude n'avait été menée à bien pour identifier les sources des épidémies et les trajets utilisés par le choléra pour diffuser dans les provinces du Kivu. Nous montrons ici que quelques villes, situées sur les rives du lac Kivu et du lac Tanganyika, jouent le rôle de sources principales pour ces épidémies et que le nombre de cas de choléra a tendance à augmenter en saison des pluies. Nous avons aussi montré que seule une minorité de déplacements de population est suivie d'épidémies de choléra. En outre, nous estimons que le faible nombre de cas de choléra notifié après l'éruption du Nyiragongo constitue un argument supplémentaire pour mettre en oeuvre des programmes destinés à restaurer et si possible à améliorer l'accès à l'eau potable dans les suites d'une catastrophe naturelle.

Manuscrit

Introduction

De nombreux facteurs ont été suspectés d'augmenter le risque d'épidémies de choléra dans une région donnée où le choléra est déjà présent. Les principaux facteurs environnementaux identifiés sont les fortes pluies, les périodes de prolifération du plancton, et l'augmentation de la température de surface de la mer[1].Toutefois, la plupart des études concernent le littoral maritime et il très peu de données existent sur les facteurs environnementaux impliqués dans la récurrence du choléra à l'intérieur des terres. Dans ce contexte, une étude récente a montré que les régions lacustres étaient le point de départ d'épidémies répétées au Katanga, une province située au sud-ouest de la République Démocratique du Congo (RDC)[2]. Des épidémies meurtrières ont aussi été observées lors de certaines crises humanitaires, plus précisément appelées urgence complexes, définies comme des « crises humanitaires dans un pays, une région ou une société dans lequel existe une dégradation absolue ou considérable de l'autorité, à cause d'un conflit interne ou externe, et qui requièrent une réponse internationale dépassant le mandat ou les capacités des programmes nationaux ponctuels et/ou au long cours des Nations Unies (ONU)[3] ». Enfin, probablement par analogie avec les urgences complexes, le risque d'épidémie de choléra est souvent mis en avant au décours des grandes catastrophes naturelles[4].

Les provinces du Nord et du Sud Kivu, au bord du lac Kivu, à l'est de la RDC, présentent une exceptionnelle accumulation de ces facteurs de risque. Elles ont été le théâtre de nombreux événements dramatiques, invasion et occupation par des forces étrangères, guerre civile, déplacements de population, éruption volcanique majeure et tremblements de terre. D'après une étude récente sur la mortalité en RDC, on estime que les conflits et crises humanitaires qui ont ravagé l'est de la RDC y ont causé la mort de 5,4 millions de personnes depuis 1998 et continuent à y occasionner au moins 45 000 décès chaque mois[5]. Malgré cette succession d'événements tragiques survenus dans les provinces du Kivu, un système de surveillance épidémiologique a été instauré à la fin des années 90 et est resté fonctionnel, enregistrant les données sur le choléra et les autres maladies transmissibles.

Nous présentons ici une description des aspects épidémiologiques du choléra dans les provinces du Kivu et une analyse de l'influence de facteurs environnementaux et géographiques, des urgences complexes et des désastres sur les épidémies de choléra. Ce travail, associé à celui précédemment effectué sur les épidémies de choléra au Katanga et au Kasaï Oriental[2], constitue la première étape d'un plan de lutte contre le choléra en RDC.

Matériel et méthodes

De janvier 2000 à décembre 2007, le nombre de cas et de décès par choléra a été recueilli chaque semaine dans chaque zone de santé (ZS) des provinces du Nord et du Sud Kivu. Un cas de choléra a été défini, suivant les recommandations de l'Organisation Mondiale de la Santé (OMS), comme « toute personne âgée de cinq ans ou plus qui développe une déshydratation sévère ou meurt à cause d'une diarrhée aqueuse aiguë », avec un âge limite abaissé à deux ans en cas d'épidémie confirmée[6]. Toujours suivant les recommandations de l'OMS, tout nouvel et important épisode de choléra a été confirmé par culture et identification de *Vibrio cholerae* O1 à partir d'échantillons de selles de malades[6].

Le Nord Kivu (53 855 km², 4 667 699 habitants, 19 ZS) et le Sud Kivu (65 000 km², 4 670 699 habitants, 14 ZS) sont situés dans le Grand Rift Africain bordées par les lacs Edouard et Kivu et par la partie nord du lac Tanganyika. Le climat de cette région est caractérisé par une saison des pluies, d'octobre à la fin mai, et une saison sèche le reste du temps. Toutefois, la saison des pluies est atténuée par une courte période presque sèche en janvier et février. Le relief dominant de ces provinces est constitué de chaînes volcaniques. Le Nyiragongo, le volcan le plus actif, se situe à environ 20 km au nord de la ville de Goma (400 000 habitants), près du lac Kivu. La dernière éruption majeure du Nyiragongo a eu lieu le 17 janvier 2002 ; date où des flots de lave ont détruit un tiers de la ville de Goma[7], faisant 147 victimes (D. Bompangue, données personnelles). En réponse à cette catastrophe, la communauté internationale a apporté une aide massive et rapide à la population et a assuré l'approvisionnant en eau potable. De plus, pendant les douze semaines suivant l'éruption, l'accès aux soins a été amélioré grâce à l'action humanitaire. En particulier, un programme d'approvisionnement en médicaments a couvert tous les centres de soins de santé primaires de Goma et les soins y ont été dispensés gratuitement pendant 6 semaines, puis à coût réduit pendant encore six semaines (0,2 $ au lieu de 1 $ par consultation, médicaments inclus). Les données sur les crises humanitaires qui sont survenues entre 2000 et 2007 ont été obtenues sur le site Reliefweb[7], qui compile des informations de sources très variées, en particulier, d'organisations non-gouvernementales (ONG) ou d'agences des Nations-Unies. Parmi les crises humanitaires signalées, nous n'avons conservé que celles qui ont fait l'objet d'une évaluation de terrain par des organisations humanitaires et qui ont impliqué plus de 1000 personnes déplacées internes.

Un système d'information géographique a été créé, basé sur les données collectées dans les 33 ZS des deux provinces. En suivant une procédure déjà décrite ailleurs [2], nous avons étudié les relations statistiques entre le nombre de cas de choléra dans chaque ZS et des

variables géographiques et environnementales (superficie, population, et présence/absence de : ville de plus de 100 000 habitants, port de commerce, axe routier majeur, situation en bord de lac), testant différents modèles de régression linéaire généralisée (quasi-poisson, distribution binomial négative de type I ou de type II) afin de tenir compte de la sur-dispersion des données. Nous avons sélectionné chaque variable suivant une démarche pas à pas et les meilleurs modèles ont été comparés en fonction des critères Akaike en se référant aux ouvrages de Venables et Ripley[8] et de Rigby et coll.[9]. Les relations entre le nombre de cas de choléra dans les zones de santé et les variables géographiques ont finalement été modélisées à l'aide d'un modèle binomial négatif de type II (fonction logarithmique pour à la fois la moyenne et la distribution des paramètres). La spatialisation des résidus a ensuite été examinée sur des variogrammes empiriques construit sur la base de distances établies en fonction des coordonnées géographiques du centroïde de chaque zone de santé. Une enveloppe de variogrammes a été construite en réalisant 1000 permutations des valeurs résiduelles des localisations spatiales, permettant une comparaison entre les limites de l'enveloppe et le variogramme. Dans notre étude, nous n'avons pas mis en évidence d'autocorrélation spatiale résiduelle.

Les taux d'attaque de choléra ont été cartographiés pour les 33 DS à l'aide de shapefiles ESRI®. L'existence de corrélations croisées entre les séries temporelles des ZS a été recherchée[10]. Les séries temporelles synchrones, sans décalage de temps au sein d'une aire géographique, ont été regroupées, définissant 5 zones (zone 1 : Mutwanga ; zone 2 : Goma, Kirotsche ; zone 3 : Bukavu, Katana ; zone 4 : Uvira, Nundu, Fizi ; zone 5 : Pinga, Walikale). Les séries temporelles obtenues ont été décomposées en tendance inter-annuelle, composante saisonnière, et résidu. Cette décomposition a fait appel à une régression de loess l'aide du progiciel MASS de R[11] qui se base sur les travaux de Cleveland et al. (1990). Dans l'étude des séries temporelles cette approche présente l'avantage d'être simple et robuste, mais elle ne permet pas de prédire et de quantifier de manière détaillée les paramètres des séries temporelles (ce qui n'était pas nécessaire pour remplir les objectifs de la présente étude). Les résidus ont été analysés et, chaque semaine, les zones ayant un nombre de cas supérieur à la moyenne ont été considérées comme étant en réactivation épidémique. Dans l'hypothèse où les réactivations épidémiques sont favorisées par les conflits, on devrait observer plus de réactivations épidémiques pendant les 12 semaines suivant un conflit que pendant les 12 semaines suivant une quelconque période de paix tirée au sort. Cette hypothèse a été testée sur la base de 1000 simulations, à la recherche d'au moins une (ou de l'absence totale de) réactivation d'épidémie pendant les 12 semaines suivant chacune des périodes tirées au sort.

Les crises humanitaires survenues alors qu'une épidémie de choléra était déjà en cours ont été exclues de cette analyse. Nous avons aussi tenu compte pour cette randomisation de la fréquence des conflits dans chacune des zones considérées. L'impact de l'éruption du Nyiragongo a aussi été analysé en relation avec la dynamique des épidémies de choléra. Il faut noter que pendant les 12 semaines qui ont suivi l'éruption du Nyiragongo, une étude épidémiologique avait été entreprise dans les centres de santé primaires de Goma. Pendant cette période, tous les cas de diarrhée aiguë, d'infection respiratoire haute ou basse et de fièvre avaient été systématiquement notés dans cinq centres de santé de Goma Ouest. Ces données ont pu être comparées à celles obtenues dans ces mêmes centres pendant les trois semaines ayant précédé l'éruption.

Les calculs et les représentations graphiques ont été faits à l'aide d'ArcGIS 8.3®, de R 7.2[12] et des progiciels complémentaires suivants: MASS[8], maptools[13], sp[14], GAMLSS[15] et GeoR[16].

Résultats

De janvier 2000 à décembre 2007 (416 semaines), un total de 73 605 cas et 1612 décès de choléra (létalité 2,2%) ont été rapportés pour le Nord et le Sud Kivu. *Vibrio cholerae* O1 El Tor Ogawa a été isolé de 8 prélèvements de selles au Nord Kivu (sur 38 prélèvements) et 3 au Sud Kivu (sur 29 prélèvements). *Vibrio cholerae* O1 El Tor Inaba n'a été trouvé qu'au Sud Kivu (6 prélèvements positifs sur 29). A Bukavu (Sud Kivu), les sérotypes Ogawa et Inaba ont été retrouvés lors d'une même épidémie de choléra en 2005. Toutes les isolats étaient sensibles à la ciprofloxacine, l'érythromycine et l'acide nalidixique, et résistantes à la tétracycline, l'ampicilline et au cotrimoxazole.

Pendant les huit ans de l'étude, les deux provinces ont subi au minimum un épisode de choléra chaque année, avec des pics variant de 130 à 700 cas par semaine (Figure 1). Au Sud Kivu, des cas de choléra ont été rapportés toutes les semaines sauf lors de deux courtes périodes en 2001 et 2002 (Figure 1). La décomposition en séries temporelles y montrait une influence saisonnière marquée, avec un plus grand nombre de cas pendant la saison des pluies. Au Nord Kivu, nous n'avons pas observé de période sans choléra. Les aspects saisonniers étaient notablement différents par rapport au Sud Kivu, avec des épidémies survenant à la fois en saison sèche et en saison des pluies (Figure 1). Des périodes de rémission partielle furent parfois observées, au cours desquelles l'incidence hebdomadaire de la maladie était inférieure à 1/50 000 habitants (en 2001, les semaines 29, 30, 31, 32, 35 et 40 ; en 2004, les semaines 23 et 28). Chaque fois, les épidémies de choléra réapparurent,

reprenant à partir de cas résiduels à Goma (Nord Kivu) ou Uvira (Sud Kivu). La distribution spatiale des cas de choléra était hétérogène, avec un grand nombre de cas dans les ZS situés en bordure de lac, alors que deux ZS excentrées, Kaziba et Shabunda, n'ont reporté aucun cas de choléra (Figure 2). Le tableau 1 montre que le nombre de cas de choléra était significativement plus élevé en cas de localisation en bordure de lac (odds ratio [OR] 7,0 ; intervalle de confiance à 95% [95% CI] 3,8-12,9).

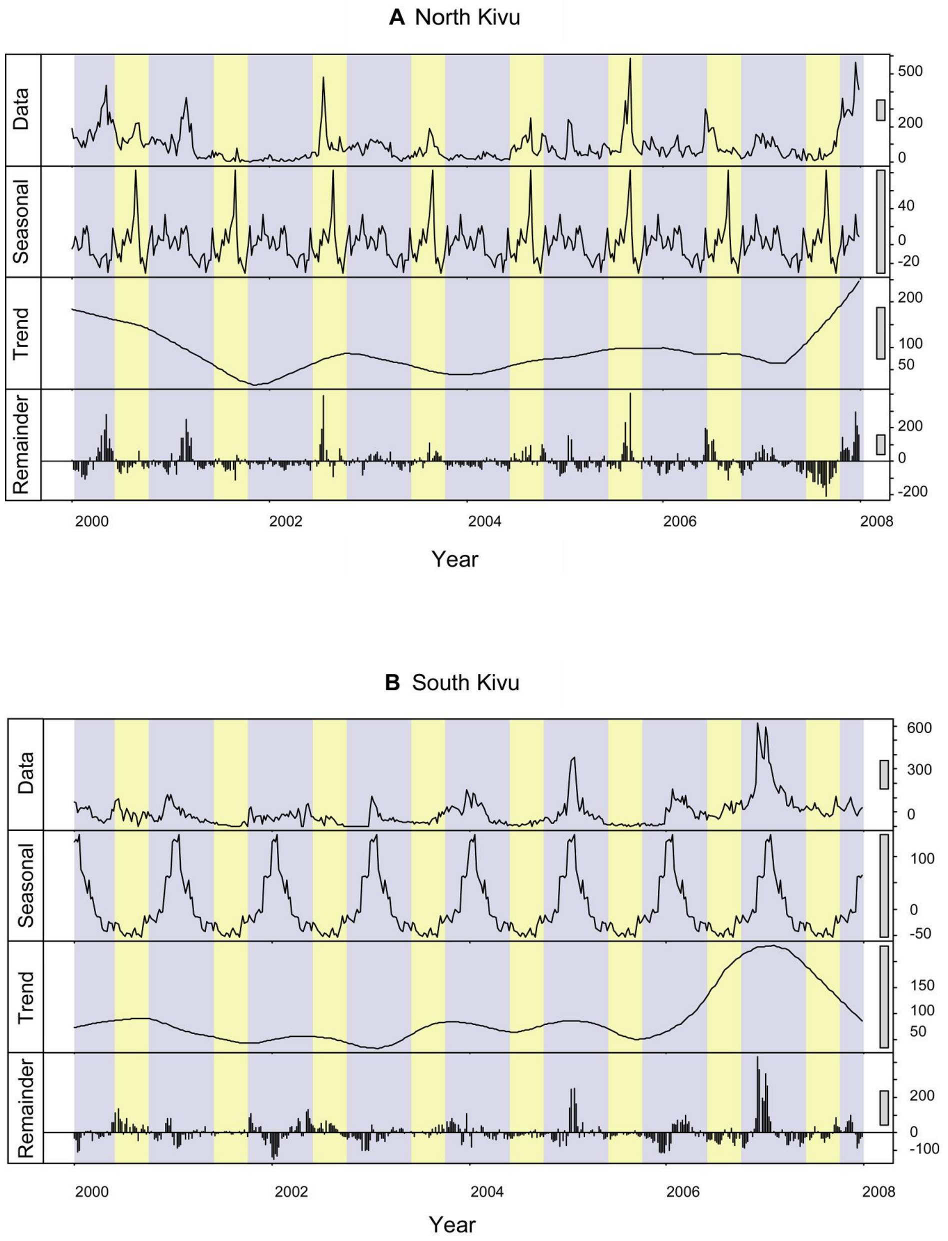

<u>Figure 1 : Variations temporelles du nombre de cas hebdomadaires de choléra de 2000 à 2007</u>.

A : Nord Kivu et **B :** Sud Kivu ; en jaune : saison sèche, en bleu : saison des pluies.

Les séries sont décomposées en saisonnalité, tendance générale et résidu.

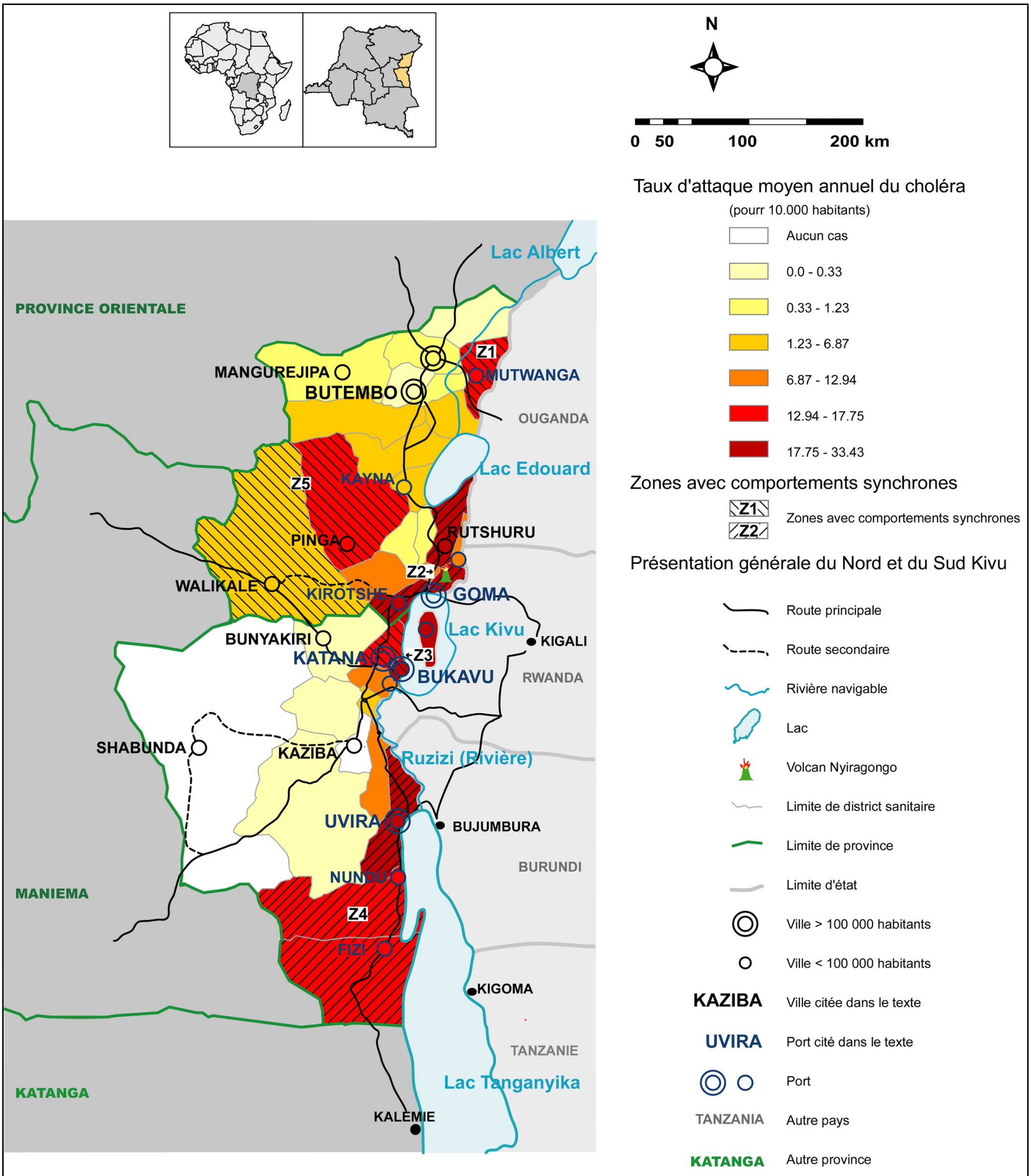

Figure 2 : Distribution du choléra au Nord et au Sud Kivu de 2000 à 2007.

Taux d'attaque annuel moyen par zone de santé pour 10 000 habitants.

Tableau 1 : Paramètres et odds-ratio du modèle binomial négatif sélectionné.

Caractéristique	Risque relatif	Limite inférieure 95%CI[1]	Limite supérieure 95%CI[1]
Intercept[2]	0,001	0,001	0,002
Ville de plus de 100 000 habitants	1,464	0,746	2,873
Port	1,608	0,687	3,765
Lac	7,015	3,813	12,904

Cas de choléra au Nord Kivu et au Sud Kivu, RDC, 2000–2007.

[1] 95% CI, intervalle de confiance à 95%;

[2] Intercept, nombre moyen de cas par zone de santé.

D'après les rapports de l'ONU et des ONG, 18 crises humanitaires accompagnées de déplacements de population de grande envergure ont été comptabilisées pendant cette période. Dans six cas, les déplacements de population sont survenus alors qu'une épidémie de choléra avait déjà commencé. Parmi les douze autres crises avec déplacement de population, quatre furent suivis d'une épidémie de choléra, qui débuta dans les douze semaines. Deux de ces épidémies eurent lieu dans des camps de déplacés internes ; elles débutèrent six à huit semaines après l'arrivée des premiers déplacés dans le camp. Toutefois, les simulations montrent que le nombre des réactivations épidémiques n'est pas plus important que celui obtenu pour n'importe quelle période sans conflit (Figure 3).

En 2002, l'éruption du Nyiragongo ne fut pas suivie d'une augmentation de l'incidence du choléra. Une étude menée dans cinq centres de santé primaires de l'ouest de Goma a montré que, durant cette période, les diarrhées représentaient seulement 6% des patients venus y consulter. De janvier à avril 2002, il y eut seulement 180 cas de choléra (8 par semaine) déclarés dans l'ensemble de la ville de Goma, sans aucun décès. Ce faible nombre de cas contraste avec la moyenne de 29 cas par semaine habituellement observée à Goma à cette période de l'année.

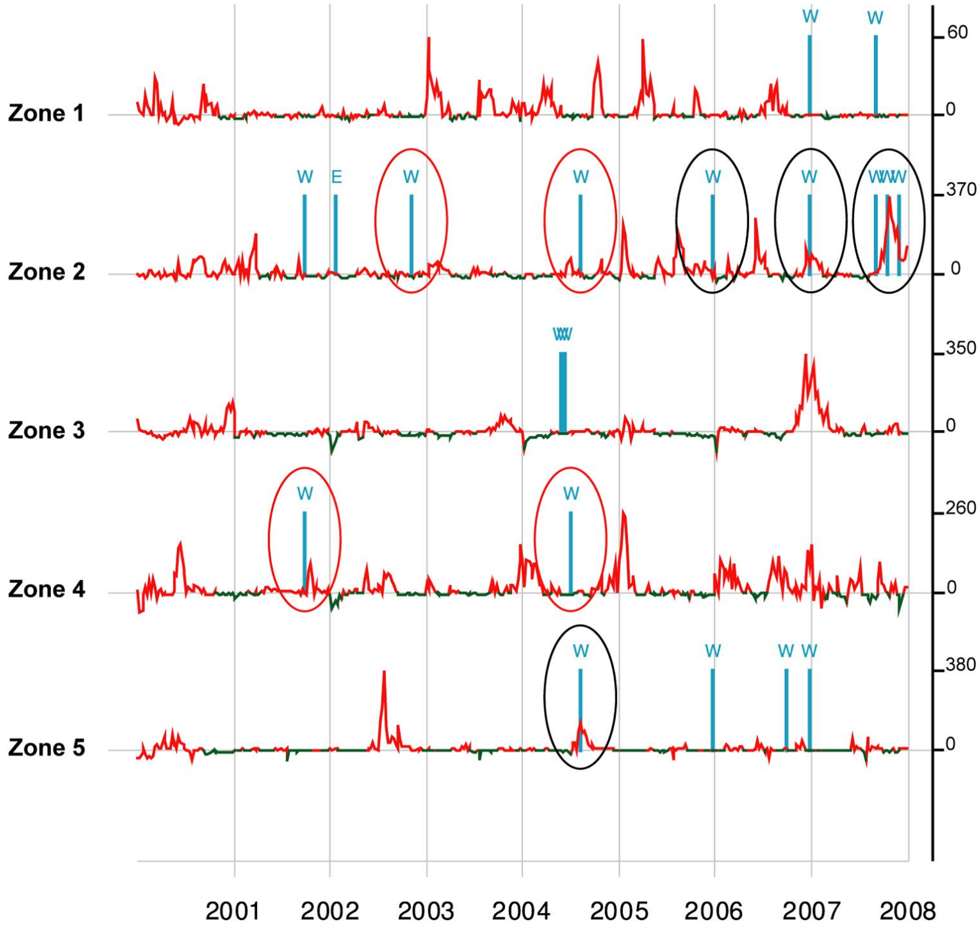

<u>Figure 3 : Epidémies de choléra et principaux événements dans les cinq ensembles de zones de santé où les épidémies se déroulaient de manière synchrones.</u>

Les conflits (W) et la principale catastrophe naturelle (l'éruption du Nyiragongo, E) observés au Nord et au Sud Kivu de 2000 à 2007.

Zone 1 : Mutwanga; Zone 2: Goma, Rutshuru, Kirotshe; Zone 3: Bukavu, Katana

Zone 4: Uvira, Nundu, Fizi; Zone 5: Pinga, Walikale.

Seuls les résidus des séries temporelles sont montrés pour éliminer les effets saisonniers et inter-annuels (en rouge : les périodes où ils sont plus élevés que la moyenne, en vert, celles où ils le sont moins).

Les conflits survenant dans une période déjà épidémique sont entourés en noir, ceux suivis dans les douze semaines par une épidémie sont entourés en rouge.

Discussion

Depuis le début des années 90, les provinces du Kivu ont constitué l'un des foyers de choléra les plus actifs du monde. En 1994, les camps de réfugiés localisés autour de Goma et de Bukavu ont été le lieu des pires épidémies de choléra observées ces cent dernières années. L'épidémie explosive qui a touché les réfugiés Rwandais, a touché environ 70 000 personnes et causé 12 000 décès[17]. Depuis lors, les épidémies sont banales dans les provinces du Kivu, et, dans une revue sur les épidémies de choléra signalées dans le monde entier de 1995 à 2005, Griffith et al. ont montré que les provinces de l'est de la RDC comptent maintenant parmi les zones les plus affectées d'Afrique[18]. En dépit des limitations inhérentes à la collecte de données épidémiologiques dans les pays en voie de développement, et plus encore dans un contexte de guerre civile, notre étude est la première à fournir des données spatialisées hebdomadaires sur une période de huit ans pour une population d'environ dix millions de personnes vivant dans une région sévèrement touchée par le choléra. Même si le nombre de cas rapportés dans cette étude est impressionnant, il est certainement sous-estimé, car l'insécurité prévalant dans cette région durant notre étude limitait fortement l'accès aux soins. Inversement, l'on ne peut pas exclure que des patients souffrant d'autres causes de diarrhées aient pu être comptabilisés dans cette étude. En effet, seul un faible nombre de diagnostics cliniques a pu être confirmé biologiquement, même en considérant que cela puisse être dû à une procédure de prélèvement inadaptée (prélèvements collectés dans des seaux contenant des résidus d'eau de Javel) ou bien à des délais d'acheminement excessifs vers le laboratoire. De plus, du fait de l'absence de moyens diagnostiques appropriés, les autres causes de diarrhées aqueuses n'ont malheureusement pu être recherchées. Par exemple, au Bangladesh, certaines épidémies de diarrhée aqueuses sont en réalité dues à des ETEC (*Escherichia coli* entéro-toxinogènes) dont la présentation clinique peut être comparable à celle des cas de choléra présentés ici[19]. En conséquence, nos résultats, basés sur une définition clinique des cas, ne doivent être considérés que comme une estimation du poids réel du choléra dans les provinces du Kivu. En dépit de toutes ces limites, nous pensons que nos résultats mettent en évidence certains aspects importants de l'épidémiologie du choléra qui méritent d'être discutés.

Tout d'abord, ces résultats, obtenus dans une région lacustre, confirment ce qui a récemment été observé dans la province du Katanga , située au sud du Kivu, dans l'est de la RDC[2]. Cette étude montrait que 60% des cas qui observés au Katanga et au Kasaï Oriental entre 2002 et 2005 étaient survenus dans un petit nombre de zones de santé bordant des lacs.

Dans ces deux provinces, le nombre de cas de choléra était significativement plus élevé en présence d'un lac (OR : 7,5 ; 95% CI : 3,9-14,2). Cette même tendance est observée dans les provinces du Kivu. Parallèlement au rôle joué par Kalemie et Bukama au Katanga, les villes de Goma, Bukavu et Uvira paraissent constituer les principales sources des épidémies de choléra dans les provinces du Kivu. D'un point de vue opérationnel, ces résultats imposent de porter une plus grande attention à ce qui se passe dans ces villes, surtout au tout début d'une épidémie, lorsque le nombre de cas est encore faible, mais commence à croître.

L'influence des saisons, et les effets que quelques zones lacustres ont sur la persistance de la maladie, rapprochent les aspects épidémiologiques du choléra au Kivu de ceux observés et bien analysés sur les côtes asiatiques [1,20,21]. Deux études ont souligné le lien entre les épidémies de choléra et le fait d'habiter sur les bords d'un lac ou d'une rivière, d'en boire l'eau et d'y laver ses affaires. Une de ces études a été menée à Rumonge, au Burundi, au bord du lac Tanganyika, non loin de la ville congolaise d'Uvira[22]. L'autre concernait une zone à proximité du lac Victoria au Kenya et soulignait que le lac pouvait servir de réservoir environnemental au moins transitoire pour le choléra et évoquait le rôle possible des jacinthes d'eau dans la persistance de vibrion toxigéniques dans l'environnement en période inter-épidémique[23]. Toutefois, les biotopes lacustres diffèrent de l'eau saumâtre des estuaires qui constituent le réservoir naturel de *V. cholerae*[24-26]. Même si l'eau des lacs est parfois riche en plancton[27], le rôle de ces lacs comme réservoir pérenne de *V. cholerae* toxigénique n'est pas établi formellement dans la mesure où aucune étude n'a jusqu'ici montré leur présence à long terme dans les lacs de la Vallée du Rift en Afrique de l'Est. Notre étude montre qu'il est nécessaire de mener des études établissant le rôle joué par les lacs dans la persistance du choléra dans les pays non côtiers d'Afrique. En effet, quelques souches endémiques de *V. cholerae* ont déjà été isolées de l'eau douce[28] et Kirschner et coll. Ont récemment montré l'existence de souches de V. cholerea non toxigéniques capable de se multiplier sous la forme de bactéries libres dans un lac naturel d'Autriche[29].

L'éruption du Nyiragongo, la seule catastrophe naturelle importante rapportée durant notre période d'étude, n'a pas été suivie d'épidémie de choléra, ni d'ailleurs d'aucune autre épidémie. Au contraire, pendant les mois qui ont suivi la catastrophe, l'incidence du choléra a été parmi les plus basses observées à Goma pendant les huit années de l'étude. Diverses hypothèses peuvent être évoquées pour expliquer ce faible nombre de cas de choléra. Cela peut être le fruit du programme d'urgence, mais d'autres explications ne peuvent être

formellement écartées, comme le fait que l'éruption volcanique a pu par elle-même diminuer le risque d'épidémie de choléra par le biais d'une modification des caractéristiques des ressources en eau et de l'usage que la population en a fait. Nos résultats sont en accord avec une étude récente qui montre que les tremblements de terre, tsunamis et éruptions volcaniques ne sont généralement pas suivis d'épidémies[30]. En particulier, depuis 20 ans, aucune épidémie de choléra n'a été observée au décours d'une catastrophe géophysique, même après le terrible tsunami asiatique de 2004. Nous confirmons ici que, même dans une région où les épidémies de choléra sont choses courantes, dans une période favorable à ces épidémies (la saison des pluies), et dans un contexte de catastrophe qui a détruit environ 12 000 habitations et une grande partie du réseau d'eau de cette ville de 400 000 habitants, la survenue d'une épidémie de choléra n'était pas inéluctable.

L'étude de l'impact des crises humanitaires nous a montré qu'elles ne sont pas un facteur déclenchant systématique des épidémies de choléra. Toutefois, dans cette étude, nous avons aussi observé quatre flambées de choléra au décours de crises humanitaires, avec, pour d'eux d'entre elles, leur survenue dans un camp quelques semaines après l'arrivée des premières personnes déplacées. De fait, plusieurs conditions doivent se trouver réunies pour qu'une crise humanitaire se complique de la survenue d'une épidémie de choléra. Parmi ces conditions, il faut que certaines des personnes déplacées ayant fui un conflit soient porteuses du germe (asymptomatique ou en incubation), et/ou que ces personnes arrivent dans une zone où il est déjà présent. Et il faut encore une absence ou une insuffisance de l'assistance aux réfugiés (fourniture d'eau potable et système de soins gratuits) pour qu'il puisse se développer. Ces conditions existaient à Goma en 1994, lorsqu'un million de réfugiés venus du Rwanda se sont installés dans des camps improvisés autour de Goma, submergeant les capacités de réponse des équipes humanitaires présentes sur place. Plus récemment, à cause de l'insécurité qui prévalait dans la région de Goma dans l'été 2008, une cascade de flambées de choléra a débuté à Rutshuru au nord de Goma et s'est étendue vers le sud et l'ouest : Rutshuru (début de l'épidémie lors de la semaine 37), Goma et Karisimbi (début semaine 40), Walikale et Birambizo (début semaine 44). Dans chacun de ces ZS, l'épidémie a été apportée par des personnes ayant fui les zones de combat du nord de Goma (D. Bompangue, données personnelles). Simultanément, à cause du danger excessif sur le terrain, de nombreuses ONG ont quitté Goma et les zones voisines, alors que le choléra débutait, laissant les programmes de lutte désorganisés.

Pour conclure, les aspects épidémiologiques du choléra dans les deux provinces du Kivu confirment nos résultats précédents au Katanga et au Kasaï Oriental, et mettent en exergue le rôle de quelques villes situées au bord d'un lac comme sources des épidémies de choléra. Cette étude montre aussi que, même si toutes les crises humanitaires suivies d'importants déplacements de population ne sont pas systématiquement accompagnées d'épidémies de choléra, ces situations d'urgence, peuvent favoriser la diffusion de la maladie, du fait de la migration de porteurs de germes depuis des foyers d'origine, vers de nouvelles zones où les ONG ne peuvent pas toujours agir à cause du risque encourus par les volontaires oeuvrant sur le terrain. En revanche, même dans un contexte d'urgence complexe ou de catastrophe naturelle, la survenue d'une épidémie est évitable. Par exemple, le nombre de cas de choléra s'est avéré étonnement bas après la destruction partielle de la ville de Goma par le volcan Nyiragongo suivie de la mise en œuvre d'une intervention humanitaire d'urgence. Nous pensons que ce faible nombre de cas est un argument de plus pour encourager la mise en œuvre de projets recherchant à restaurer et si possible à améliorer l'accès à une eau potable dans les suites d'une catastrophe naturelle.

Remerciements

Nous remercions le Ministère de la Santé de RDC, les équipes médicales congolaises du Nord Kivu et du Sud Kivu pour leur collaboration.

Sources de financement

Cette étude a obtenu le soutien financier partiel de l'Université de Franche-Comté, de la Région de Franche-Comté et de l'IMEA, Institut de Médecine et d'Epidémiologie Appliquées – Fondation Léon Mba, et de la Fondation Veolia Environnement. Ces organismes n'ont joué aucun rôle dans la construction de l'étude, ni dans la collecte ou l'analyse de données, ni dans la preparation ou la publication du manuscrit.

Références

1. Huq A, Sack RB, Nizam A, Longini IM, Nair GB, et al. (2005) Critical factors influencing the occurrence of Vibrio cholerae in the environment of Bangladesh. Appl Environ Microbiol 71: 4645-4654.
2. Bompangue D, Giraudoux P, Handschumacher P, Piarroux M, Sudre B, et al. (2008) Lakes as source of cholera outbreaks, Democratic Republic of Congo. Emerg Infect Dis 14: 798-800.
3. Spiegel PB, Le P, Ververs MT, Salama P (2007) Occurrence and overlap of natural disasters, complex emergencies and epidemics during the past decade (1995-2004). Confl Health 1: 2.
4. Watson JT, Gayer M, Connolly MA (2007) Epidemics after natural disasters. Emerg Infect Dis 13: 1-5.
5. Coghlan B, Brennan RJ, Ngoy P, Dofara D, Otto B, et al. (2006) Mortality in the Democratic Republic of Congo: a nationwide survey. Lancet 367: 44-51.
6. WHO (1996) Guidelines for cholera control. Genova: WHO. Available: http://whqlibdoc.who.int/publications/1993/924154449X.pdf. Accessed 12 april 2009.
7. Telesmanich NR, Koliakina AV, Lomov Iu M, Men'shikova EA, Mironova AV (2008) [Characterization of the adhesive activity of cholera vibrions in mammalian red blood cells as an additional test for assessment of their epidemic significance]. Klin Lab Diagn: 45-48.
8. Venables WN, Ripley BD (2002) Modern Applied Statistics with S. New York: Springer Science+Business Media.
9. Rigby RA, Stasinopoulos DM (2005) Generalized additive models for location, scale and shape, (with discussion). . Applied Statistics 54: 507-554.
10. Legendre P, Legendre L (1998) Numerical ecology. Amsterdam: Elsevier. 870 p.
11. Cleveland RB, Cleveland WS, McRae JE, Terpenning I (1990) STL: A Seasonal-Trend Decomposition Procedure Based on Loess. Journal of Official Statistics 6: 3-73.
12. R Development Core Team (2008) R: A language and environment for statistical computing. Vienna: R Foundation for Statistical Computing. Avalaible: http://cran.r-project.org/doc/manuals/refman.pdf. Accessed 12 april, 2009.
13. Lewin-Koh NJ, Bivand R (2008) Tools for reading and handling spatial objects. pp. Avalaible: http://cran.r-project.org/web/packages/maptools/index.html. Accessed 12 april 2009.
14. Pebesma EJ, Bivand R (2005) Classes and methods for spatial data in R. R-news 5: 9-13. Available: http://cran.r-project.org/doc/Rnews/Rnews_2005-2.pdf. Accessed 12 april 2009.
15. Stasinopoulos DM, Rigby B (2007) Generalized Additive Models for Location Scale and Shape (GAMLSS) in R. Journal of Statistical Software 23: 1-46. Available: http://www.jstatsoft.org/v23/i07/paper. Accessed 12 april 2009.
16. Ribeiro PJ, Diggle PJ (2001) geoR: A package for geostatistical analysis. R-news 1: 15-18. Available: http://cran.rproject.org/doc/Rnews/Rnews_2001-2.pdf. Accessed 12 april 2009.
17. Siddique AK, Salam A, Islam MS, Akram K, Majumdar RN, et al. (1995) Why treatment centres failed to prevent cholera deaths among Rwandan refugees in Goma, Zaire. Lancet 345: 359-361.
18. Griffith DC, Kelly-Hope LA, Miller MA (2006) Review of reported cholera outbreaks worldwide, 1995-2005. Am J Trop Med Hyg 75: 973-977.

19. Harris AM, Chowdhury F, Begum YA, Khan AI, Faruque AS, et al. (2008) Shifting prevalence of major diarrheal pathogens in patients seeking hospital care during floods in 1998, 2004, and 2007 in Dhaka, Bangladesh. Am J Trop Med Hyg 79: 708-714.
20. Koelle K, Rodo X, Pascual M, Yunus M, Mostafa G (2005) Refractory periods and climate forcing in cholera dynamics. Nature 436: 696-700.
21. Sack RB, Siddique AK, Longini IM, Jr., Nizam A, Yunus M, et al. (2003) A 4-year study of the epidemiology of Vibrio cholerae in four rural areas of Bangladesh. J Infect Dis 187: 96-101.
22. Birmingham ME, Lee LA, Ndayimirije N, Nkurikiye S, Hersh BS, et al. (1997) Epidemic cholera in Burundi: patterns of transmission in the Great Rift Valley Lake region. Lancet 349: 981-985.
23. Shapiro RL, Otieno MR, Adcock PM, Phillips-Howard PA, Hawley WA, et al. (1999) Transmission of epidemic Vibrio cholerae O1 in rural western Kenya associated with drinking water from Lake Victoria: an environmental reservoir for cholera? Am J Trop Med Hyg 60: 271-276.
24. Colwell RR (2004) Infectious disease and environment: cholera as a paradigm for waterborne disease. Int Microbiol 7: 285-289.
25. Ramamurthy T, Yamasaki S, Takeda Y, Nair GB (2003) Vibrio cholerae O139 Bengal: odyssey of a fortuitous variant. Microbes Infect 5: 329-344.
26. Tantillo GM, Fontanarosa M, Di Pinto A, Musti M (2004) Updated perspectives on emerging vibrios associated with human infections. Lett Appl Microbiol 39: 117-126.
27. Plisnier P-D, Chitamwebwa D, Mwape L, Tshibangu K, Langenberg V, et al. (1999) Limnological annual cycle inferred from physical – chemical fluctuations at three stations of Lake Tanganyika. Hydrobiologia 407: 45-58.
28. Islam MS, Drasar BS, Sack RB (1994) The aquatic flora and fauna as reservoirs of Vibrio cholerae: a review. J Diarrhoeal Dis Res 12: 87-96.
29. Kirschner AK, Schlesinger J, Farnleitner AH, Hornek R, Suss B, et al. (2008) Rapid growth of planktonic Vibrio cholerae non-O1/non-O139 strains in a large alkaline lake in Austria: dependence on temperature and dissolved organic carbon quality. Appl Environ Microbiol 74: 2004-2015.
30. Floret N, Viel JF, Mauny F, Hoen B, Piarroux R (2006) Negligible risk for epidemics after geophysical disasters. Emerg Infect Dis 12: 543-548.
